# Supplementary material for: P-TRAP: a Panicle Trait Phenotyping tool
Source: BMC Plant Biol. 2013 Aug 29;13:122. doi: 10.1186/1471-2229-13-122 (PMC3848748; doi:10.1186/1471-2229-13-122)
Supplement: Additional file 7 — P-TRAP source code. The Netbeans project that contains the source code of the application. [file 1471-2229-13-122-S7.zip › javadoc.zip!/javadoc/org-basmati-workspace/allclasses-frame.html]

All Classes (WorkSpace)


# All Classes

- CollectMasterReportAction
- ColoredImageAction
- Drawer
- EditandViewGrainFileActionDataObject
- EditResultFileAction
- EditViewGrains
- ExpressRunAction
- FindGrainsAction
- FindGrainsSingleImageAction
- FindGrainTraitsAction
- FindGrainTraitsSingleImage
- FindSkeletonAction
- FindStructureSingleImageAction
- GrainsFinder
- GrainTraitsFinder
- GraphEditorTopComponent
- GraphUI
- GraphUI.calcType
- ImageScaler
- ImageTypeAction
- ImageViewScene
- ImportImages
- OpenImageRice
- ParticleScene
- ParticlesEditorTopComponent
- ProcessedRiceImageFileDataObject
- ProjectUIManager
- ReportGenerator
- ResultProducer
- ResultsEditorTopComponent
- ScaleImage
- ScaleSelectorPanel
- ScalingPanel
- StructureFinder
- VeiwParticlesAnchors
